# Cysteine cathepsins control hepatic NF-*κ*B-dependent inflammation via sirtuin-1 regulation

**DOI:** 10.1038/cddis.2016.368

**Published:** 2016-11-10

**Authors:** Álvaro de Mingo, Estefanía de Gregorio, Anna Moles, Núria Tarrats, Anna Tutusaus, Anna Colell, Jose C Fernandez-Checa, Albert Morales, Montserrat Marí

**Affiliations:** 1 Department of Cell Death and Proliferation, IIBB-CSIC/IDIBAPS, Barcelona, Catalonia, Spain; 2 Fibrosis Research Group, Institute of Cellular Medicine, Newcastle University, Newcastle Upon Tyne, UK; 3 Research Center for Alcoholic Liver and Pancreatic Diseases, Keck School of Medicine of the University of Southern California, Los Angeles, CA, USA

## Abstract

Sirtuin-1 (SIRT1) regulates hepatic metabolism but its contribution to NF-*κ*B-dependent inflammation has been overlooked. Cysteine cathepsins (Cathepsin B or S, CTSB/S) execute specific functions in physiological processes, such as protein degradation, having SIRT1 as a substrate. We investigated the roles of CTSB/S and SIRT1 in the regulation of hepatic inflammation using primary parenchymal and non-parenchymal hepatic cell types and cell lines. In all cells analyzed, CTSB/S inhibition reduces nuclear p65-NF-*κ*B and *κ*B-dependent gene expression after LPS or TNF through enhanced SIRT1 expression. Accordingly, SIRT1 silencing was sufficient to enhance inflammatory gene expression. Importantly, in a dietary mouse model of non-alcoholic steatohepatitis, or in healthy and fibrotic mice after LPS challenge, cathepsins as well as NF-*κ*B-dependent gene expression are activated. Consistent with the prominent role of cathepsin/SIRT1, cysteine cathepsin inhibition limits NF-*κ*B-dependent hepatic inflammation through the regulation of SIRT1 in all *in vivo* settings, providing a novel anti-inflammatory therapeutic target in liver disease.

Hepatic inflammation is a central part of the liver response to injury elicited by diverse harmful stimuli such as alcohol, cholestasis, and excess fat, among others.^1^ The essential function of the liver for homeostasis and inflammatory responses is emphasized by its anatomical location, allowing continuous blood flow from the gastrointestinal tract through the hepatic sinusoids. This allows hepatocytes and non-parenchymal cells to interact with gut-derived pathogens and their products, such as LPS.^1^ In this interplay, the regulation of hepatic functions is greatly facilitated by cytokines, and both LPS and the LPS-induced cytokine TNF play a central role in liver homeostasis and inflammation through the activation of NF-*κ*B transcription factor in different liver cell populations. NF-*κ*B is a homo- or heterodimeric complex formed by Rel-like domain-containing proteins, the p65-p50 complex being the most abundant.

The activation of NF-*κ*B leads to the transcription of genes with “healthy” or “unhealthy” function depending on the cell type,^2^ encoding for cytokines, chemokines, and also genes coding for regulators of apoptosis and cell proliferation.^3^ Since unrestrained inflammation may be harmful, NF-*κ*B is regulated at several levels, including controlled cytoplasmic-nuclear shuttling and modulation of its transcriptional activity.^3^ In unstimulated cells, NF-*κ*B is held in the cytoplasm complexed to its repressor I*κ*B. In a conventional, or canonical, activation pathway, NF-*κ*B needs first to be freed from I*κ*B. To this aim, I*κ*B is phosphorylated by I*κ*B kinases in response to activators such as LPS or TNF, and subsequently degraded, thus liberating the active NF-*κ*B complex, which translocates to the nucleus. The post-translational regulation of the active NF-*κ*B subunits is mainly achieved by phosphorylation and acetylation of p65 at specific residues^2^ that can either enhance NF-*κ*B activity, by controlling p65 recruitment binding to *κ*B DNA binding sites, or ending gene transcription by inducing its nuclear export.^4^ The responsible enzymes for these modifications are emerging but are not well understood.^2^

Sirtuin-1 (SIRT1), a prototype mammalian NAD(+)-dependent protein deacetylase, has emerged as a key metabolic sensor in various metabolic tissues. Hepatic SIRT1 controls several transcription factors that regulate glucose, lipid, and cholesterol homeostasis.^5^ Actually, liver-specific SIRT1 knockout mice develop hepatic steatosis,^6^ and extensive weight loss in obese patients increases sirtuin expression significantly in both adipose tissue and liver, probably as a consequence of reduced inflammation.^7^ Regarding inflammation, it has been described that NF-*κ*B is a target of SIRT1. In their seminal study in 2004, Yeung *et al.*^8^ demonstrated that SIRT1 could directly deacetylate the p65 at lysine 310 residue, thus inhibiting the transactivation capacity of p65 and suppressing NF-*κ*B-dependent gene transcription.

Cathepsin B (CTSB), a ubiquitous cysteine cathepsin, is a lysosomal/endosomal protease whose participation in different pathologies such as liver fibrosis,^9^ atherosclerosis,^10^ Alzheimer disease,^11^ and cancer^12^ has been reported in the past years. Moreover, CTSB has been implicated in hepatic lysosomal disruption and apoptosis.^13, 14, 15^ Interestingly, CTSB is also localized in the nucleus, where it is found to be associated with the nuclear scaffold.^16^ Interestingly, the proteolytic cleavage of SIRT1 by cysteine cathepsins, CTSB, and Cathepsin S (CTSS), among others, has been observed in osteoarthritic chondrocytes and endothelial progenitor cells.^17, 18^

Of note, in previous studies in murine models of toxic-induced liver fibrosis, we observed that CTSB inhibition mitigated chronic CCl_4_-induced inflammation, hepatic stellate cell (HSC) activation, and collagen deposition.^9^ Similarly, other studies using both pharmacological and genetic CTSB inactivation observed reduced hepatic inflammation, as assessed by transcripts for CXC-chemokines and neutrophil infiltration after cholestasis.^19^ However, in both studies, the mechanism behind the pro-inflammatory role of CTSB was lacking.

Given the central role of NF-*κ*B in hepatic inflammation and the recognized participation of SIRT1 in liver homeostasis, we wondered if there exists a connection between SIRT1 and NF-*κ*B in hepatic inflammation, and if cysteine cathepsins were somehow modulating this outcome. To this aim, we analyzed in different liver cell types if physiological effectors of hepatic damage and inflammation, such as TNF or LPS, through NF-*κ*B, were affected by cysteine cathepsin inhibition, and if SIRT1 was involved in this process.

## Results

### In HSCs NF-*κ*B-dependent gene-expression is modulated by SIRT1 through CTSB/S

**Figure 1:**
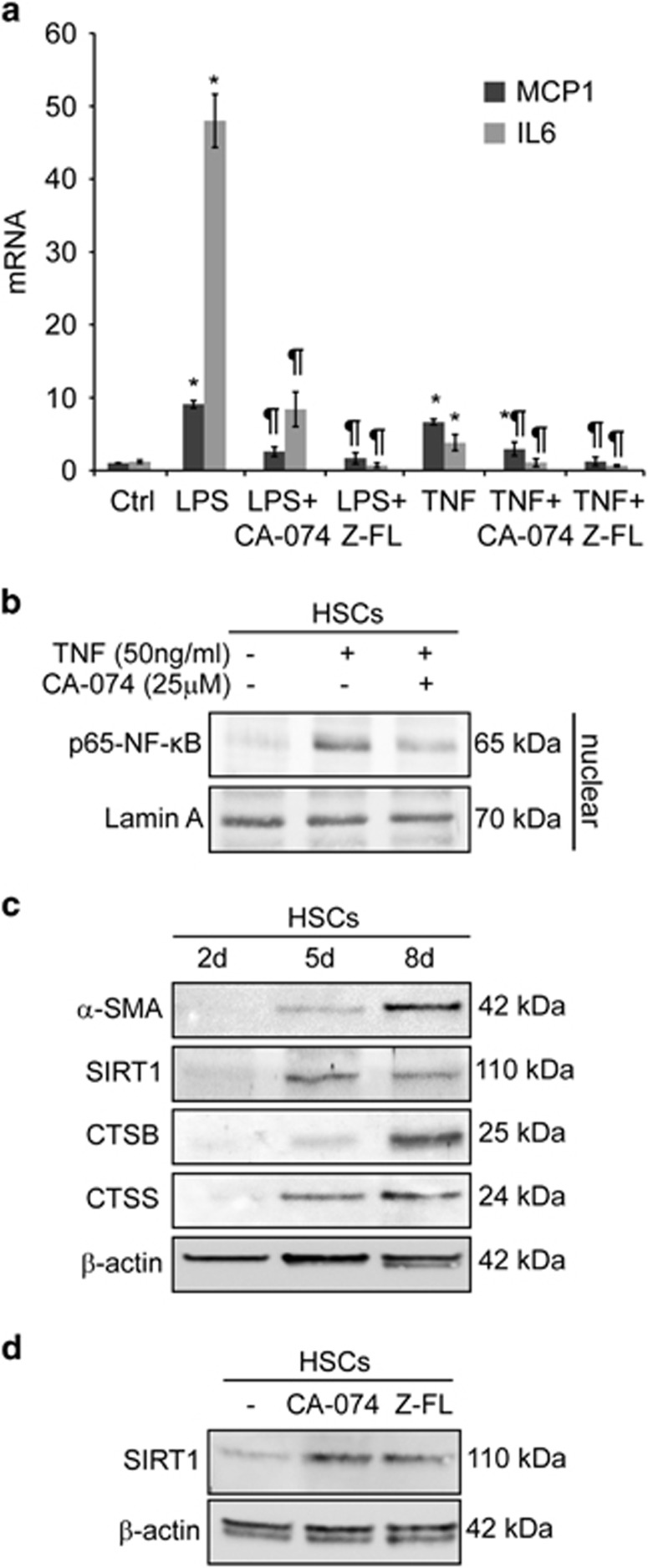
Cysteine cathepsins modulate SIRT1 and NF-*κ*B induction in primary HSCs. (**a**) MCP1 and IL6 mRNA expression in primary HSCs (8-days) preincubated with CA-074 (25 *μ*M) or Z-FL (10 *μ*M) overnight, and exposed to TNF (50 ng/ml) or LPS (50 ng/ml) for 6 h. (**b**) Immunoblots displaying nuclear extracts of HSCs (8-days) treated with CA-074, overnight, and challenged with TNF for 10 min. (**c**) *α*-SMA, SIRT1, CTSB, and CTSS protein expression during primary HSC transdifferentiation. (**d**) Expression of SIRT1 in HSCs (8-days) exposed to CA-074 or Z-FL, overnight. Results are given as mean±S.D., *n*=8; *P<0.001 *versus* Control, and ^¶^*P*<0.001 *versus* LPS or TNF treatment

**Figure 2:**
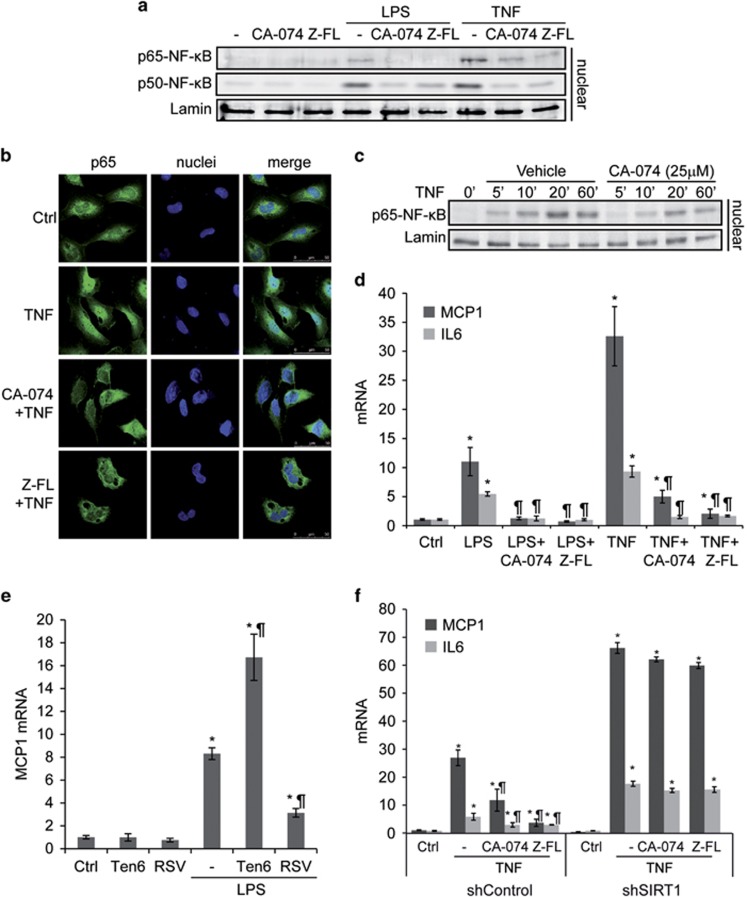
Regulation of NF-*κ*B-dependent gene expression by CTSB and CTSS in LX2 cells. (**a**) Immunoblot of nuclear extracts, or (**b**) p65-NF-*κ*B confocal microscopy of LX2 cells exposed to CA-074 (25 *μ*M) or Z-FL (10 *μ*M) overnight, and challenged for 10 min TNF (50 ng/ml), or after 30 min LPS (50 ng/ml). (**c**) Time course of p65-NF-*κ*B in nuclear extracts of LX2 cells exposed to TNF with/without preincubation with CA-074 (25 *μ*M, overnight). (**d**) mRNA expression of MCP1 and IL6 in LX2 cells after overnight preincubation with CA-074 or Z-FL followed by exposure to TNF or LPS for 6 h. (**e**) MCP1 mRNA expression in LX2 cells preincubated overnight with Ten6 (10 *μ*M) or RSV (100 *μ*M) followed by 6 h LPS. (**f**) MCP1 and IL6 mRNA expression in LX2 cells infected with Control or SIRT1 shRNA, preincubated with CA-074 (25 *μ*M) or Z-FL (10 *μ*M) followed by exposure to 6 h TNF. Results are given as mean±S.D., *n*=4; *P<0.05 *versus* control or shControl, and ^¶^*P*<0.05 *versus* LPS- or TNF-treated cells

To evaluate the role of cysteine cathepsins in NF-*κ*B-dependent inflammatory gene expression, mouse primary activated HSCs (8-days) were challenged with LPS or TNF. Both mediators induced an enhancement of the *κ*B-dependent MCP1 chemokine, and IL6 cytokine mRNAs. Preincubation of HSCs with CA-074-methyl-ester or Z-FL-COCHO, CTSB and CTSS inhibitors, respectively,^20, 21^ greatly reduced TNF- or LPS-induced MCP1 and IL6 mRNA (Figure 1a). Consistent with this, in activated primary HSCs, p65-NF-*κ*B nuclear translocation upon TNF challenge was reduced in cells after CTSB inhibition (Figure 1b). In addition, CTSB, as expected,^9^ and also CTSS increased their expression during the HSC activation process. Of note, in primary HSCs, SIRT1's gradual increase during activation was suppressed in fully activated HSCs (Figure 1c), coinciding with the maximum cathepsin expression. In fact, SIRT1 levels increased after CTSB or CTSS inhibition (Figure 1d), thus supporting the cleavage of SIRT1 by cysteine cathepsins in HSCs, as described previously in other cell types.^17, 18^ Of interest, in the human HSC cell line LX2,^22^ LPS or TNF administration resulted in p65-NF-*κ*B and p50-NF-*κ*B, nuclear translocation that was blunted upon preincubation with CTSB or CTSS inhibitors (Figure 2a). Confocal microscopy also revealed that p65-NF-*κ*B nuclear presence was reduced by Cathepsin B or S (CTSB/S) inhibition after TNF challenge (Figure 2b). Moreover, CTSB inhibition reduced overall p65-NF-*κ*B nuclear presence, induced by TNF, at all time-points analyzed (Figure 2c). In addition, CTSB or CTSS inhibitors, by themselves, did not alter MCP1 or IL6 expression, or induced p65-p50-NF-*κ*B translocation, as compared to untreated HSCs or LX2 cells (not shown); however they were able to greatly diminish the enhanced MCP1 and IL6 mRNA expression induced by TNF or LPS (Figure 2d).

To determine if SIRT1 expression could modulate the NF-*κ*B-dependent response, LX2 cells were preincubated with SIRT1 activator (Resveratrol, RSV) or inhibitor (Tenovin-6, Ten6), and exposed to LPS. As shown in Figure 2e, LPS-induced MCP1 mRNA expression was diminished upon SIRT1 activation and enhanced after its antagonism. Similarly, in LX2 cells infected with SIRT1 shRNA, stable SIRT1 decrease (~90% SIRT1 mRNA decline) led to superior MCP1 and IL6 mRNA expression (Figure 2f) upon TNF exposure, as compared to control shRNA-infected LX2 cells. Of note, while CTSB or CTSS inhibition in control shRNA LX2 cells induced a decrease in MCP1 and IL6 mRNA expression, *versus* TNF only, this outcome was not observed in SIRT1 shRNA clones (Figure 2f), therefore implicating that SIRT1's presence is required for cathepsins to affect NF-*κ*B-dependent transcription.

### NF-*κ*B-dependent gene expression is modulated by CTSB/S in hepatocytes

**Figure 3:**
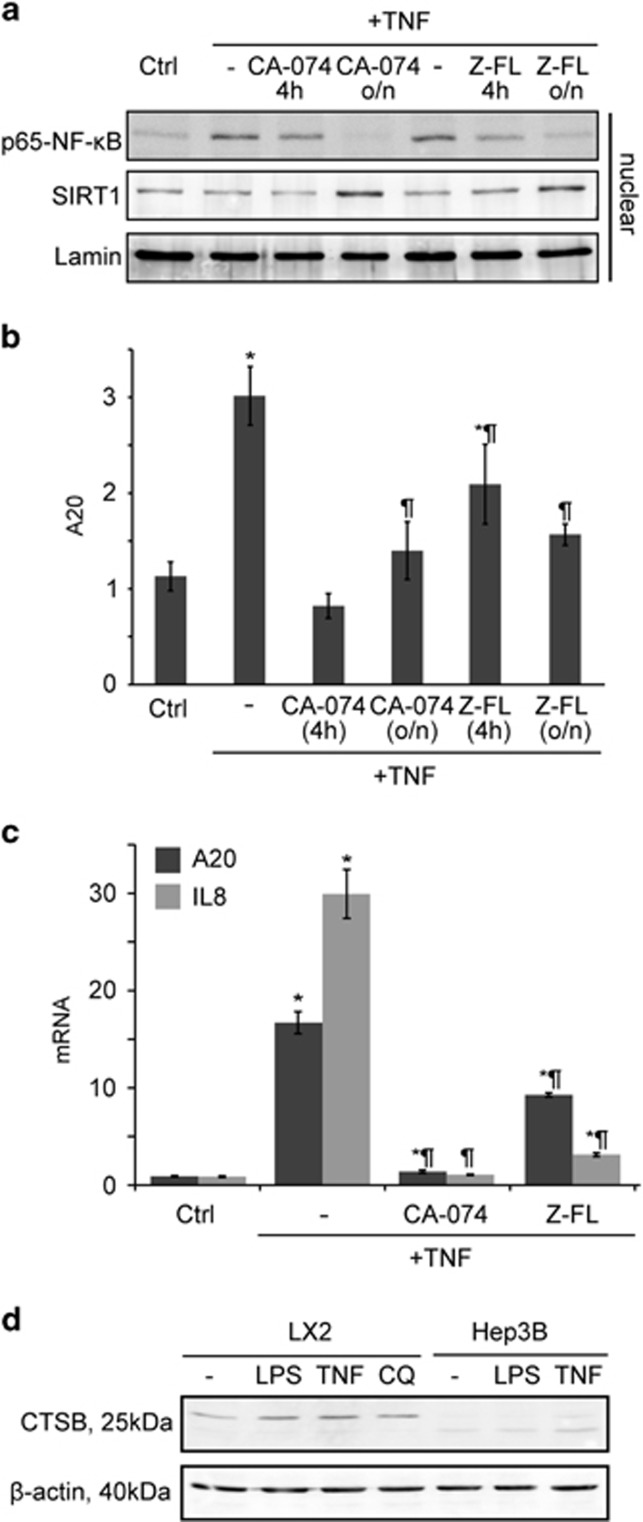
Effect of CTSB/S inhibition on NF-*κ*B activation in hepatocytes. Primary mouse hepatocytes were exposed to CA-074 (25 *μ*M) or Z-FL (10 *μ*M), overnight or for 4 h. (**a**) Nuclear protein levels were analyzed after 10 min TNF challenge. (**b**) A20 mRNA expression was quantified after 6 h TNF challenge. (**c**) A20 and IL8 mRNA expression in Hep3B cells pre-treated overnight with CA-074 (25 *μ*M) or Z-FL (10 *μ*M) before 6 h TNF challenge. (**d**) Immunoblot of CTSB in the cytosolic fraction after challenging LX2 or Hep3B cells with LPS, TNF or chloroquine (CQ, 50 *μ*M) for 60 min. Results are given as mean±S.D., *n*=5; *P<0.001 *versus* control, and ^¶^*P*<0.001 *versus* TNF

We next evaluated whether a similar relationship was present in hepatocytes. Primary mouse hepatocytes were treated with TNF, and p65 nuclear translocation and the expression of the NF-*κ*B-dependent antiapoptotic protein A20 were analyzed. TNF-induced translocation of p65-NF-*κ*B to the nucleus was decreased upon CTSB or CTSS inhibition, accompanied by an increase in nuclear SIRT1 expression (Figure 3a). Moreover, p65 translocation correlated with NF-*κ*B-dependent expression of A20 mRNA (Figure 3b).

We also evaluated in Hep3B cells, a human hepatoma cell line, mRNA expression of A20 and of IL8, a chemokine induced by TNF in human cell types with no murine homolog. In Hep3B cells, TNF-induced A20 and IL8 mRNA expression levels were greatly reduced after CTSB or CTSS inhibition (Figure 3c). Moreover, in LX2 cells treated with LPS, TNF, or chloroquine (a lysosomotropic agent, used as a positive control) we observed the release of CTSB to the cytosol in LX2, but only after TNF challenge in Hep3B, consistent with their low TLR4 activity (Figure 3d).

### In macrophages, NF-*κ*B-dependent inflammatory response is controlled by CTSB/S

**Figure 4:**
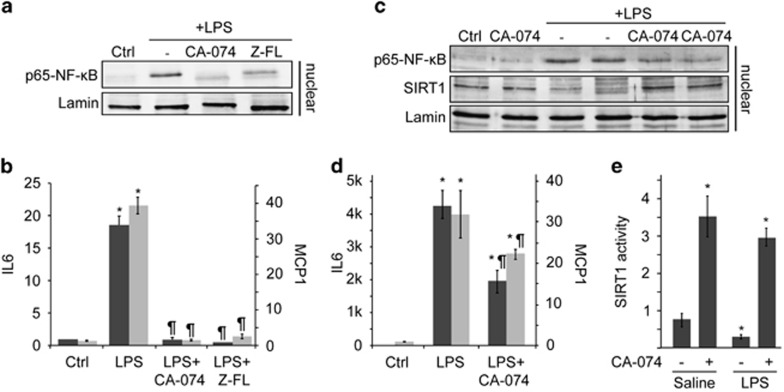
NF-*κ*B-dependent response to LPS is controlled by cathepsins in macrophages. KCs were exposed overnight to CA-074 (75 *μ*M) or Z-FL (7.5 *μ*M) and (**a**) nuclear proteins were analyzed after LPS (50 ng/ml, 30 min) challenge, or (**b**) IL6 and MCP1 mRNA expression was analyzed after 6 h LPS. RAW264.7 cells were pre-treated with CA-074 (75 *μ*M, 4 h) and (**c**) nuclear proteins analyzed after LPS challenge (50 ng/ml, 30 min), or (**d**) MCP1 and IL6 mRNA expression was analyzed after 6 h LPS. (**e**) Total SIRT1 activity was determined in RAW264.7 cells pre-treated with CA-074 (75 *μ*M, overnight) and exposed to LPS (30 min). Results are given as mean±S.D., *n*=4; *P<0.001 *versus* Control, and ^¶^*P*<0.05 *versus* LPS

We next moved to primary Kupffer cells (KCs), since LPS-mediated KC activation is a key mechanism in the pathogenesis of various liver diseases.^1^ In KCs, LPS exposure induced p65-NF-*κ*B nuclear translocation accompanied by a prominent MCP1 and IL6 mRNA induction that was blunted after CTSB or CTSS inhibition (Figures 4a and b). We next corroborated our observations in RAW264.7, murine macrophage cells, observing parallel results (Figures 4c and d). Both in KCs and RAW264.7 cells, the concentration of CTSB inhibitor was increased to 75 *μ*M in order to achieve optimal CTSB inhibition (>90%). CTSS inhibition in RAW264.7 cells resulted in cell death; therefore it was not used for this set of experiments. Of interest, in RAW264.7 cells, LPS induced a decrease in SIRT1-110 kDa that was recovered after CTSB inhibition (Figure 4c). This result was corroborated by measuring SIRT1 activity (Figure 4e). Of note, it is interesting to observe that while CTSB inhibition *per se* did not affect either p65-NF-*κ*B translocation, or MCP1 and IL6 mRNA expression (not shown), it did enhance SIRT1 activity in RAW264.7 cells independent of LPS treatment (Figure 4e).

### The processing of SIRT1 correlated with CTSB activity and the inflammatory phenotype

**Figure 5:**
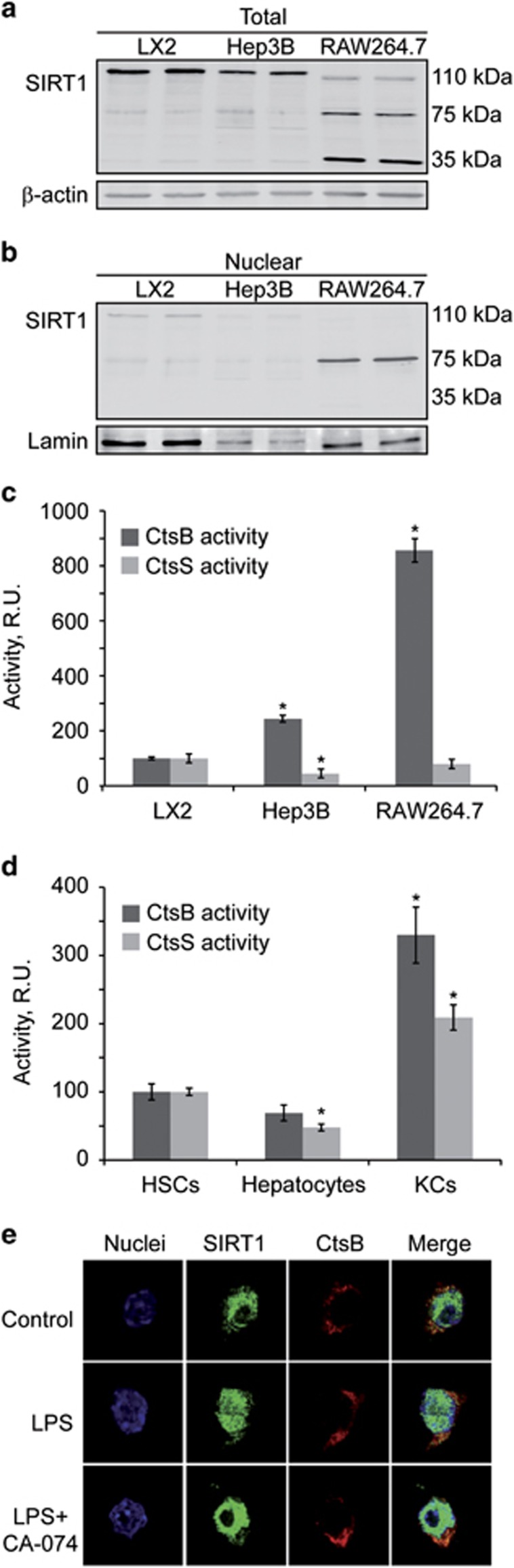
SIRT1 correlates with CTSB activity in cell lines and primary cells. (**a**) Total and (**b**) nuclear SIRT1 expression; and (**c**) CTSB and CTSS activities in LX2, Hep3B and RAW264.7 cells. (**d**) CTSB and CTSS activity in HSCs (8-days old), hepatocytes, and KCs. (**e**) SIRT1 and CTSB confocal microscopy of RAW264.7 macrophages pretreated with CA-074 (75 *μ*M, 4 h) before LPS challenge (50 ng/ml, 30 min). Representative image, *n*=3. Results are given as a mean±S.D., *n*=5; *P<0.001 compared to LX2 or HSCs values

In RAW264.7 cells, the SIRT1 immunoblot, apart from the full-length 110 kDa band, resolved two additional bands (75 and 35 kDa), bands that were barely visible in LX2 or Hep3B cells where the full-length protein, SIRT1-110 kDa, was most noticeable (Figure 5a). Consistent with this, in the nucleus, the active isoform of SIRT1 was present in LX2 and Hep3B cells but almost inexistent in RAW264.7 cells, where we only observed the SIRT1-75 kDa form (Figure 5b). On the other hand, when measuring CTSB activity in the different cells, we observe remarkable variations among cells. Interestingly, macrophages, both RAW264.7 cells and KCs, exhibit more CTSB activity (eight- and threefold, as compared to LX2 and HSCs respectively) than the other cell types. In contrast, CTSS activity was more stable between the three cell populations, displaying no consistent differences between phenotypes (Figures 5c and d). Therefore, as RAW264.7 cells display an inflammatory phenotype and higher levels of CTSB, we chose this cell line for analyzing SIRT1 and CTSB location by confocal microscopy. CTSB inhibition increased nuclear SIRT1 in LPS-treated cells (in green) (Figure 5e). In addition, it is important to stress that the CTSB inhibitor is inhibiting CTSB activity, but not removing CTSB protein. That is why, although inactive, CTSB levels appear increased after its inhibition. These results, together with the observation that in RAW264.7 cells, CTSB inhibition enhances SIRT1 activity (Figure 4e), suggest that the processing of SIRT1 correlates with CTSB activity, in the different cell lines analyzed, and with their potential inflammatory phenotype.

### *In vivo,* CTSB/S inhibition lessens LPS-derived NF-*κ*B-dependent inflammatory gene expression

**Figure 6:**
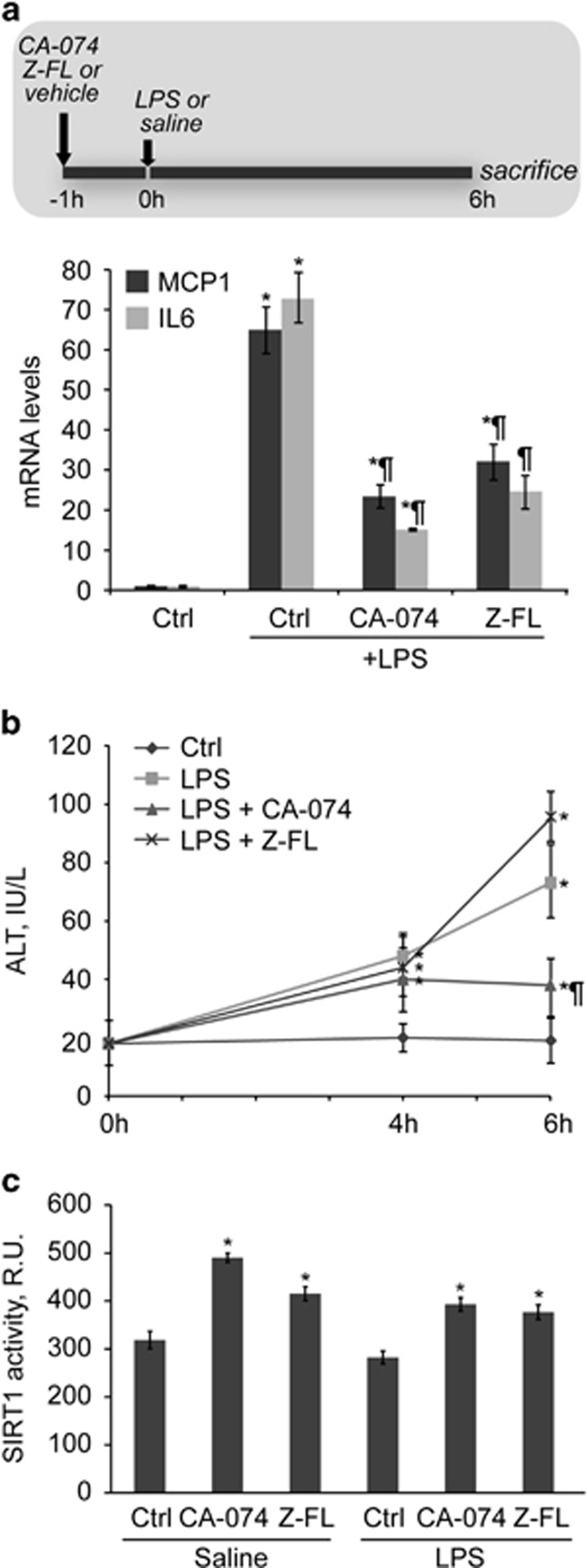
Effect of CTSB/S inhibition in an *in vivo* model of acute inflammation. Mice were treated with CTSB/S inhibitors (10 mg/kg) 1 h before LPS i.p. injection (1.0 mg/kg, 6 h) and (**a**) MCP1 and IL6 mRNA expression was determined in liver tissue, (**b**) ALT levels were measured in blood collected at 0, 4, 6 h, and (**c**) hepatic SIRT1 activity was analyzed. Results are given as a mean±S.E.M., *n*=6, *P<0.001 *versus* Control, and ^¶^*P*<0.05 *versus* LPS

To validate our observations *in vitro*, we next moved onto *in vivo* studies. First, we induced acute inflammation in healthy mice with LPS for 6 h. Neither CA-074 nor Z-FL by themselves, administered 1 h prior LPS, had any effect on liver damage or basal *κ*B-dependent gene expression and behaved like the untreated control mice (not shown). NF-*κ*B-dependent genes increased upon LPS challenge, and this induction was diminished in mice after CTSB or CTSS inhibition (Figure 6a). Treatment of mice with liposomal clodronate significantly reduced the extent of MCP1 and IL6 mRNA induction, indicating KCs as its source (Fig. S1). Of interest, LPS-induced liver damage was significantly reduced in mice receiving CTSB inhibitor, but not after CTSS inhibition (Figure 6b), suggesting, in addition to lessening inflammation, a specific role of CSTB in LPS-dependent hepatocyte cell death. Of note, both inhibitors did increase, *per se* and after LPS injection, total hepatic SIRT1 activity (Figure 6c).

**Figure 7:**
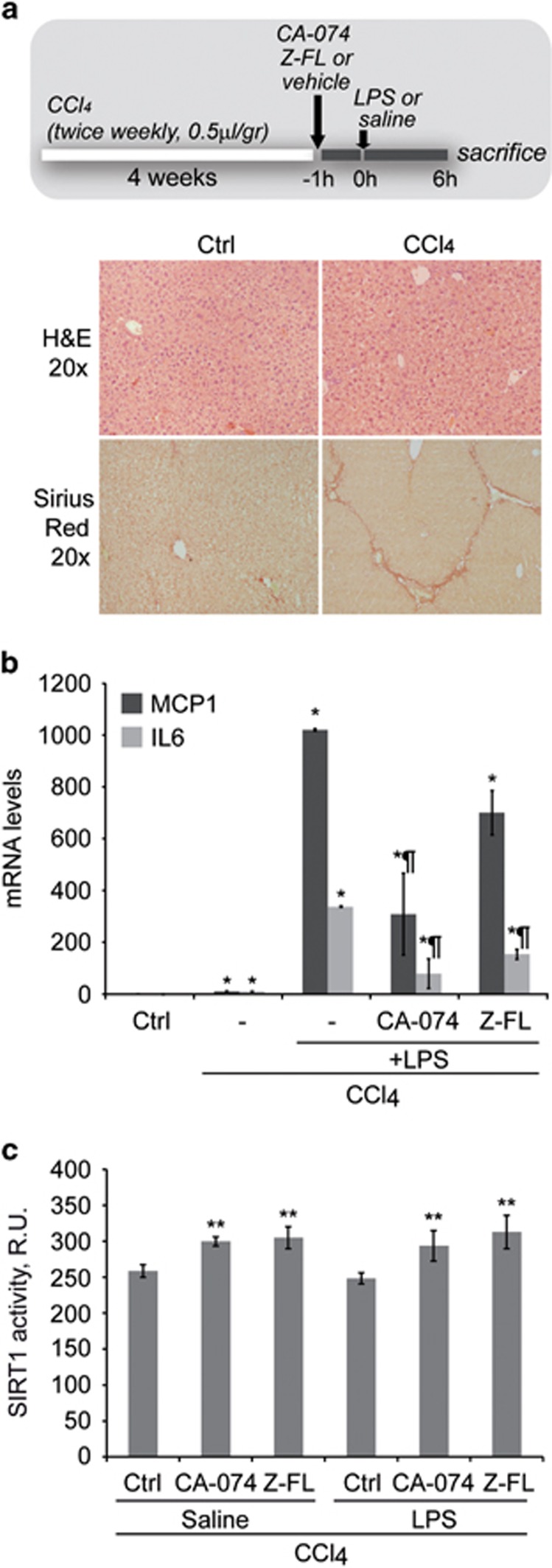
Effect of CTSB/S inhibition on liver inflammation in mice with hepatic fibrosis. (**a**) After inducing liver fibrosis in mice by CCl_4_ injection (twice weekly, 0.5 *μ*l/gr) and after H&E and Sirius Red staining displaying collagen accumulation, mice received CTSB/S inhibitors (10 mg/kg) followed 1 h later by LPS i.p. injection (1.0 mg/kg, 6 h). Then, (**b**) MCP1 and IL6 mRNA expression was determined in liver tissue, and (**c**) hepatic SIRT1 activity was analyzed. (**a**) Results are given as a mean±S.E.M., *n*=6, *P<0.001 *versus* Control, ^¶^*P*<0.001 *versus* LPS, and (**b**) **P<0.05 *versus* Ctrl with CCl_4_

Next, we evaluated CTSB/S inhibition in animals with liver fibrosis, where an important inflammatory response to LPS was expected. The experimental setup, H&E and Sirius Red staining of hepatic specimens are displayed in Figure 7a. As shown in Figure 7b, LPS-induced MCP1 and IL6 mRNA expression was much greater than in healthy mice (Figure 6a). Importantly, CTSB and CTSS inhibition significantly reduced LPS-dependent MCP1 and IL6 mRNA expression, thus confirming the effectiveness of this strategy in controlling hepatic inflammation. Again, CTSB/S inhibition resulted in a significant increase in hepatic SIRT1 activity (Figure 7c) even in LPS-treated mice, thus suggesting that enhanced SIRT1 activity results in decreased NF-*κ*B-dependent induction of inflammatory gene expression.

### CTSB inhibition reduced inflammation in a murine model of non-alcoholic steatohepatitis (NASH)

**Figure 8:**
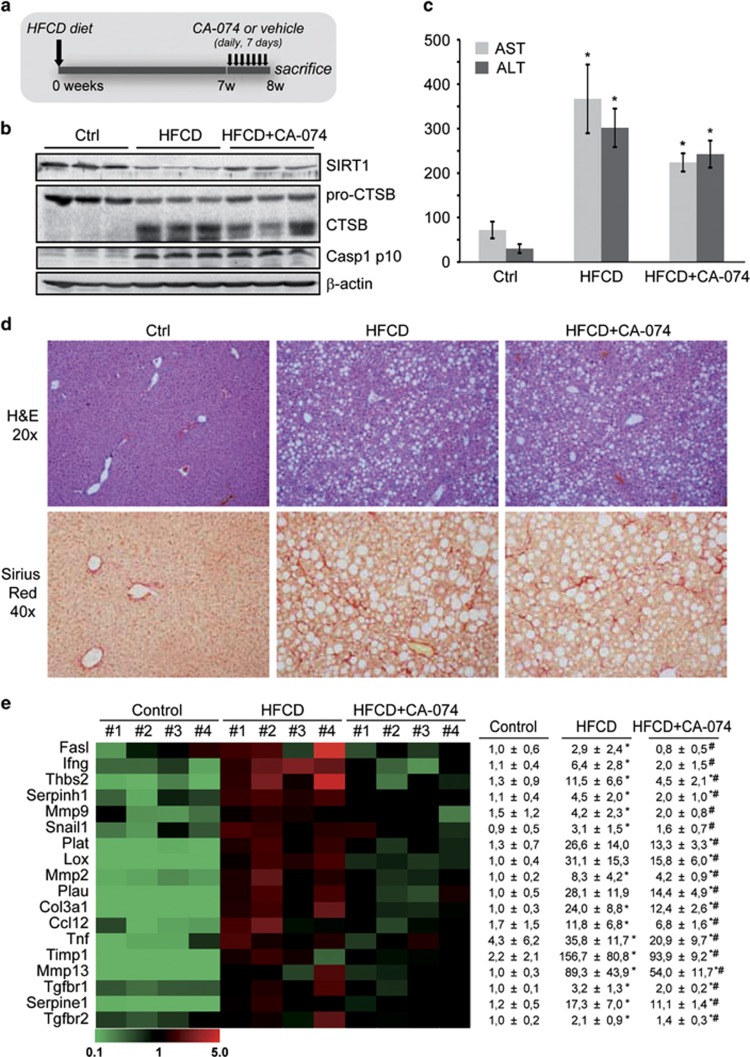
CTSB inhibition reduces inflammatory gene expression in mice with NASH. NASH was induced by feeding mice a HFCD diet for 8 weeks, CA-074-treated mice received a daily dose (10 mg/kg) for the last week. (**a**) Experimental setup, (**b**) CTSB, SIRT1 and active Caspase-1 immunoblot of hepatic lysates, (**c**) transaminases in serum, (**d**) H&E and Sirius Red staining. Results are given as mean±S.E.M., *n*=6, *P<0.01 *versus* Control. (**e**) Gene array of Control, HFCD, and HFCD+CA-074 mice displaying only those genes altered in the HFCD group that significantly improved after CA-074 treatment. For complete gene array, please refer to Figure S2

We next assessed in a dietary model of NASH (a high-fat choline-deficient (HFCD) diet) that causes macrovesicular steatosis, fibrosis, and inflammation^23^ the effect of CTSB inhibition. The experimental setup is described in Figure 8a. HFCD-feeding induced CTSB activation as observed by the processing of pro-CTSB in liver samples, and reduced full-length SIRT1 (Figure 8b). CTSB inhibition partly recovered pro-CTSB and SIRT1 levels (Figure 8b) but did not alter liver damage (Figure 8c). We also observed enhanced activation of caspase-1 in HFCD-fed mice, indicative of NLRP3-inflammasome activation, which was not altered by CTSB inhibition. H&E and Sirius Red staining are shown in Figure 8d. As expected, HFCD induced hepatic inflammation and fibrosis. The improvement in the collagen deposit observed after CTSB inhibition did not reach statistical significance (*P*=0.14, *n*=6) at the time point analyzed. However, a gene array revealed that HFCD induced important alterations in the expression of genes related to fibrosis and inflammation (i.e. FasL, IFN*γ*, MMPs, LOX, TNF, chemokines, TIMP1), many of them NF-*κ*B-dependent, that significantly improved after CTSB inhibition (Figure 8e).

## Discussion

Cathepsins are proteolytic enzymes implicated in a wide range of physiological functions, including cytosolic and nuclear functionality as revealed by several studies.^12, 24^ TNF could also destabilize lysosomal membranes, thus releasing cathepsins to the cytosol,^25^ as we observed in LX2 and Hep3B cells (Figure 3d). p65-NF-*κ*B subunit is acetylated in the nucleus, which is essential to maintain its nuclear localization and consequent NF-*κ*B transcriptional activity,^26, 27^ and it can be deacetylated by SIRT1 decreasing NF-*κ*B-dependent gene expression.^8, 28^ In activated HSCs, SIRT1 expression is inversely controlled by CTSB and CTSS activities, SIRT1 lowering being essential to establish the pro-inflammatory phenotype of activated HSCs. Our data in activated HSCs confirm the recovery of SIRT1-110 kDa after CTSB/S inhibition (Figure 1d), underscoring the role of SIRT1-CTSB/S axis in HSC activation. Of note, CTSB inhibition significantly reduces the overall presence of p65-NF-*κ*B in the nuclear fraction after TNF challenge in LX2 cells, thus suggesting that SIRT1 upregulation contributes not only to end transcription but also to diminish p65-NF-*κ*B nuclear presence. Given that SIRT1 deacetylation of p65 has been mainly associated with the ending of NF-*κ*B transcription, the observation that it can also regulate p65 nuclear export merits further investigation.^8^

SIRT1 inhibitor (Tenovin-6) and activator (Resveratrol) have opposite effects as modulators of SIRT1 activity. In our study, we observe that inflammation is enhanced when SIRT1 is inactivated by Tenovin-6, whereas cells are protected against inflammation by the use of Resveratrol (Figure 2e). These results corroborate that SIRT1 is tightly implicated in the regulation of *κ*B-dependent gene expression. To better analyze the participation of cysteine cathepsins in this process, we transfected human LX2 cells with lentiviral particles shSIRT1 and shControl. As we expected, the shSIRT1-LX2 cell line expressed a higher inflammatory phenotype compared to the shControl LX2 cells. Of interest, the employment of CTSB/S inhibitors in the shSIRT1-LX2 clone did not alter *κ*B-dependent gene expression after TNF challenge, which pointed out that in the absence of SIRT1, the inhibition of CTSB or CTSS was ineffective, therefore reinforcing the direct link between SIRT1 and cathepsins. In sum, our data indicate that cathepsin/SIRT1 modulation controls NF-*κ*B-dependent gene expression in activated HSCs, providing a novel targetable pathway to induce phenotypic changes in HSCs during liver pathology.

On the macrophage side, the expansion of our observations to KCs and RAW264.7 cells, which have a prominent inflammatory phenotype, allowed us to corroborate CTSB/S inhibition as an effective strategy to control *κ*B-dependent gene expression, under conditions where a massive release of cytokines and chemokines is induced. Interestingly, when we analyzed the expression of CTSB/S and SIRT1, we observed that RAW264.7 cells showed the highest CTSB activity, which was followed by virtually no presence of active SIRT1-110 kDa, presenting the truncated forms, SIRT1-75 kDa and SIRT1-35 kDa. In contrast, other liver cell types, such as LX2 and Hep3B cells, have predominantly the SIRT1-110 kDa full length form. More importantly, CTSB inhibition *per se* prominently recovered SIRT1 activity, by increasing it by 4-5-fold, thus emphasizing the relationship between these enzymes in macrophages. In KCs and in RAW264.7 cells higher doses of the CTSB inhibitor (75 *μ*M instead of 25 *μ*M) were required to achieve optimal CTSB inhibition (>90%), probably reflecting the high presence of this protease in macrophages. In addition, SIRT1 activity studies also revealed that upon LPS challenge there was a significant decrease in SIRT1 protein and activity in RAW264.7 cells, which could be related to the leakage of CTSB from lysosomes observed after this stimulus, suggesting that some lysosomal membrane permeabilization (LMP) may be taking place.

Finally, we tried to translate our *in vitro* observations to *in vivo* models of acute hepatic inflammation in healthy and fibrotic mice by LPS injection. In these settings, NF-*κ*B-dependent genes increased upon LPS challenge and decreased after CTSB/S inhibition, while SIRT1 activity did the opposite, hence reproducing our *in vitro* results in a more complex setup. In addition, transaminase levels indicate that CTSB inhibition, but not CTSS inhibition, lessened liver damage after LPS challenge, which could indicate again the participation of this protease in apoptotic pathways and LMP induced by TNF/LPS in hepatocytes, as described by others.^13, 14^ Of note, several groups, ours among others,^9^ have observed that antagonism of CTSB, either genetic or pharmacological, results in reduced inflammation and/or hepatic fibrosis.^9, 19, 29, 30, 31^ In these studies, the reduced inflammation observed was considered secondary to the general improvement in hepatic condition mainly attributed to CTSB's role in hepatocyte apoptosis,^15, 19, 29, 31^ or, in fibrogenesis, as a regulator of the PI3K/AKT pathway in response to PDGF in HSCs.^9^ However, our study provides direct evidence of the mechanism behind CTSB's pro-inflammatory role, as a modulator of the NF-*κ*B-inflammatory response, through SIRT1, in macrophages, HSCs, and probably in other inflammatory cells infiltrating the liver. Interestingly, we tried to attain the same goal by administering the SIRT1 agonist SRT2104 (100 mg/kg, 1 week) to mice in our CCl_4_-induced liver fibrosis model, without achieving recovery in SIRT1 activity (data not shown), and, accordingly, liver inflammation or damage was not modified by SRT2104 treatment. In contrast, the consistent increase in SIRT1 activity, both after LPS challenge in control and CCl_4_-exposed mice, and in the NASH murine model, obtained by CTSB inhibition, revealed to be a highly effective strategy to recover SIRT1 levels in the damaged liver.

Related to the role of CTSB/SIRT1 in NASH, our previous studies showed in patients with NASH, compared with healthy controls, that CTSB mRNA was increased eightfold, suggesting CTSB participation in disease progression.^32^ Additionally, hepatic SIRT1 expression is reduced not only in different animal models of NAFLD^33^ but also in patients with NASH correlating with disease severity.^34^ Of relevance, deletion of SIRT1 in mice fed a high-fat diet results in hepatic steatosis and inflammation.^6^ In our NASH model, significant changes in gene expression and overall improvement in many genes related to inflammation (FasL, IFNg, TNF, chemokines) and fibrosis (TIMP1, LOX, Col3a1, MMPs, TGFBR) is observed after CTSB inhibition in HFCD-fed mice, in parallel with increased SIRT1 expression.

Of interest, the participation of CTSB in the activation of the NLRP3 inflammasome is still debated,^35^ which contributes to enhance the inflammatory state in NASH by activating Caspase-1 and inducing the release of IL-1*β*. In this aspect, and despite CTSB being activated upon HFCD feeding, we observe enhanced Caspase-1 activation that was not affected by CTSB inhibition, not supporting a prominent role for CTSB in inflammasome activation in this setting.

Overall, our study discloses the CTSB/S-SIRT1 axis as a new route for controlling inflammation in acute and chronic hepatic diseases. In fact, given the presence of this control pathway in all hepatic cells analyzed and in RAW264.7 cells, it is possible that the importance of this mechanism reaches beyond the liver. Our data demonstrate that cysteine cathepsin inhibition limits NF-*κ*B-dependent hepatic inflammation through the regulation of SIRT1, and consequently provides an anti-inflammatory targetable pathway in liver therapy, and possibly in other organs.

## Materials and Methods

### Cell lines and cell treatment

Commercially available Hep3B, RAW264.7 cells (ECACC, Sigma-Aldrich, St. Louis, MO, USA) were used. LX2 cells were kindly given by Dr. Ramón Bataller (IDIBAPS, Barcelona, Spain). All cell lines are mycoplasma-free. Human recombinant TNF (Peprotech, Rocky Hill, NJ, USA), LPS (*E. coli* 0111:B4, Sigma-Aldrich) were administered to cells at 50 ng/ml. CTSB inhibitor (CA-074 methyl ester, Sigma-Aldrich) was given at 25 *μ*M to primary hepatocytes, HSCs, Hep3B and LX2 cells and at 75 *μ*M to KCs and RAW264.7 cells. CTSS inhibitor (Z-FL-COCHO, Calbiochem, San Diego, CA, USA) was given at 10 *μ*M to primary hepatocytes, HSCs, Hep3B and LX2 cells and at 7.5 *μ*M to KCs. Resveratrol (100 *μ*M) and Tenovin-6 (10 *μ*M) (Santa Cruz Biotechnology, Dallas, TX, USA) were given to LX2 cells. Control and human SIRT1 shRNA lentiviral particles commercially available were used (Santa Cruz Biotechnology).

### *In vivo* models

Animal studies were conducted in accordance with the principles and procedures outlined in the National Institutes of Health Guide for the Care and Use of Laboratory Animals and were approved by the institutional animal care committee of the Universitat de Barcelona. Animals were monitored daily and those with evident discomfort were euthanized, according to approved protocol. C57BL/6 male mice, 8–10 weeks old, were used. In all groups (*n*=6), animals were randomly assigned to the different groups and the liver specimens and samples obtained were blindly analyzed. Liver fibrosis was induced by injecting CCl_4_ (5 *μ*l/g of a 10% solution of CCl_4_ in corn oil) twice-weekly, for 4 weeks. Acute inflammation was induced in healthy mice by a single LPS injection (1.0 mg/kg, intraperitoneal). Treatment with CA-074-Me or Z-FL inhibitors (10 mg/kg, intraperitoneal) was initiated 1 h before LPS. In mice with fibrosis LPS was given 72 h after the last dose of CCl_4_. Mice were killed6 h after LPS. Control animals received vehicle alone. To induce NASH, mice were fed a HFCD (60% kcal) diet with 0.1% methionine (Open Source diets #A06071302) for 8 weeks, receiving daily doses of CA-074-Me (10 mg/kg, intraperitoneal) for the last week.

### Isolation and culture of hepatic parenquimal and non-parenquimal cells (NPCs)

C57BL/6 male mice, 8–12 weeks old, were from Charles River (Wilmington, MA, USA). Hepatocytes, HSCs and KCs were isolated by liver perfusion with collagenase-pronase as described^9^ with small modifications. Hepatocytes were separated from NPCs by 60 g centrifugation. The supernatant containing NPCs was collected and centrifuged at 600 g for 8 min. The NPC pellet was resuspended in 6 ml of GBSS and mixed with 6 ml of cold Nycodenz at 32% to reach 16%, then it was topped with 2 ml GBSS and centrifuged at 2300 g, 45 min, 4 °C without break. KCs appear in the interface and HSCs in the brown cloudy strip. NPCs were collected with a Pasteur tip, washed twice with GBSS at 500 g, 5 min, 4 °C and resuspended in culture media. Hepatocytes and HSCs were cultured in DMEM, and KCs in RPMI, both media complemented with 10% FBS, and antibiotics at 37°C in a humidified atmosphere of 95% air and 5% CO_2_.

### KCs elimination by clodronate injection

Commercially available suspension of liposome-encapsulated clodronate containing 5 mg of clodronate per 1 ml or vehicle (PBS) (www.clodronateliposomes.com) was given to mice by i.p. injection (dose 0.1 ml/10g mouse) 48 h before LPS.

### SDS-PAGE and immunoblot analysis

Cell lysates were prepared in RIPA buffer (50 mM Tris·HCl, pH 8, 150 mM NaCl, 1% Nonidet P-40, 0.1% SDS, 1% Triton X-100 plus proteinase inhibitors). Protein concentration was determined by Bradford assay, and samples containing 10–50 *μ*g were separated by SDS-PAGE. Proteins were transferred to nitrocellulose membranes. After this, membranes were blocked in 8% nonfat milk in 20 mM Tris-HCl, 150 mM NaCl, and 0.05% Tween-20 for 1 h at room temperature.

### Antibodies

Anti-SIRT1 (sc-15404), anti-CTSS (sc-6505), anti-p65-NF*κ*B (sc-372), anti-p50-NF*κ*B (sc-7178), anti-Lamin (sc-6215) were taken from Santa Cruz Biotechnology; anti-*β*-actin-HRP (A3854) from Sigma-Aldrich; anti-alpha smooth muscle actin (A5228) from Sigma; and anti-CTSB (06-480) from Millipore (Billerica, MA, USA).

### CTSB, CTSS and SIRT1 activities

CTSB activity was assayed fluorimetrically with Z-Arg-Arg-7-amido-4-methylcoumarin hydrochloride (60 *μ*mol/L) at pH 7.4 and 37 °C as previously described.^9^ CTSS and SIRT1 activities were determined fluorimetrically using commercially available kits (ab65307 and ab156065 respectively, Abcam, Cambridge, UK) and following the manufacturer's instructions.

### Nuclear extract isolation

2 × 10^6^ cells were scraped in Buffer A (10 mmol/L Hepes, 10 mmol/L KCl, 0.1 mmol/L ethylenediaminetetraacetic acid (EDTA), 0.1 mmol/L ethylene glycol tetraacetic acid (EGTA), 1 mmol/l dithiothreitol (DTT), and 0.5 mmol/l phenylmethylsulfonyl fluoride (PMSF), kept on ice for 15 min, lysed by the addition of 1/20 (vol/vol) 10% Igepal and vortexed for 10 s. Nuclei were pelleted (12 000 g, 30 s), resuspended in Buffer C (20 mmol/lHepes, 0.4 mol/l NaCl, 1 mmol/l EDTA, 1 mmol/l EGTA, 1 mmol/l DTT, and 1 mmol/l PMSF), and incubated for 15 min on ice with gentle mixing. Subsequently, nuclear extracts were obtained by centrifuging at 4 ºC, 12 000 g for 5 min.

### H&E and sirius red staining

Livers were formalin-fixed and 7 *μ*m sections were routinely stained with H&E or a 0.1% Sirius Red-picric solution following standard procedures. The slices were examined with a Zeiss Axioplan microscope equipped with a Nikon DXM1200F digital camera. For collagen-fiber determination series of four random selected fields from each slice were visualized and quantified using Quantity One software (Bio-Rad Laboratories, Hercules, CA, USA).

### shRNA lentiviral infection

Infection with control or hSIRT1 shRNA Lentiviral particles was performed following the manufacturer's protocol (Santa Cruz Biotechnology). Briefly, LX2 cells were grown in a 12-well plate to 50% confluency and infected with 10 *μ*l of lentiviral particles in the presence of 5 *μ*g/ml Polybrene. Forty-eight hours after infection, selection of clones was performed in complete media containing Puromycin (2 *μ*g/ml) until resistant colonies can be identified and selected.

### Laser confocal imaging

Cells were cultured on coverslips for different time-points. A 4% paraformaldehyde solution was used as a fixative agent for 15 min. When necessary cells were permeabilized with 0.1% saponine/0.5% BSA fatty acid free/PBS 1 × for 15 min, and blocked for 45 min in 3% BSA/PBS buffer. Coverslips were incubated overnight with the primary antibodies in 1% BSA/PBS buffer. Afterwards they were rinsed with PBS buffer and incubated with fluorescent secondary antibodies for 45 min. After final washes in PBS, coverslips incubated with either DAPI or DRAQ5 for 30 min in the dark to stain the nuclei and next were washed with PBS and mounted in Fluorescent Mounting Medium. Confocal images were collected using a laser scanning confocal microscope Leica DM 2500.

### RNA isolation and Real-Time RT-PCR

Total RNA was isolated with TRIzol reagent. Real-time RT-PCR was performed with iScript One-Step reverse transcription (RT)-PCR Kit with SYBR Green following the manufacturer's instructions (Bio-Rad Laboratories). The primers sequences used are described in Table 1.

### Gene array

A predesigned 384-well mouse fibrosis panel for use with SYBR Green (Bio-rad) was used following the manufacturer's instructions. Briefly, after isolating RNA with the TRIzol reagent, the corresponding cDNA was synthesized using the iScript advanced cDNA synthesis kit (Bio-rad). Once cDNA is obtained the PCR reaction mix is prepared (SsoAdvanced universal SYBR Green supermix) and added to the 384-well plate where all the primers are lyophilized. Results are corrected and normalized to the housekeeping genes *β*-actin and Tbp.

### Statistical analysis

All *in vitro* experiments were repeated at least three times. Results are expressed as mean±S.D for cell studies, and as mean±S.E.M. for *in vivo* studies. Statistical comparisons were performed using unpaired two-tailed Student's *t* test, since the samples have similar variance. All analyses were performed using GraphPad Prism. A *P* value<0.05 was considered significant.

## Figures and Tables

**Table 1:** Sequence of primers used for qPCR

**Primer description**	**Forward (5′ to 3′)**	**Reverse (5′ to 3′)**
Human MCP1	TCAAACTGAAGCTCGCACTC	ATTGATTGCATCTGGCTGAG
Human IL6	GACAGCCACTCACCTCTTCA	CCTCTTTGCTGCTTTCACAC
Human TNF	TTTGATCCCTGACATCTGGA	GGCCTAAGGTCCACTTGTGT
Human A20	ACATCCTCAGAAGGCCAATC	GCTGTCATAGCCGAGAACAA
Human IL8	AGGACAAGAGCCAGGAAGAA	ACTGCACCTTCACACAGAGC
Human *β*-actin	GGACTTCGAGCAAGAGATGG	AGGAAGGAAGGCTGGAAGAG
Mouse MCP1	CAAGAAGGAATGGGTCCAGA	GCTGAAGACCTTAGGGCAGA
Mouse IL6	CCGGAGAGGAGACTTCACAG	CCGGAGAGGAGACTTCACAG
Mouse A20	TGGGAAGGGACACAACTACA	GCAGAAACTTCCTCGTCCTC
Mouse *β*-actin	GACGGCCAGGTCATCACTAT	CGGATGTCAACGTCACACTT

## Abbreviations

CA-074: CA-074-methylester
CTSB: cathepsin-B
CTSS: cathepsin-S
KCs: Kupffer cells
HSCs: hepatic stellate cells
LMP: lysosomal membrane permeabilization
RSV: Resveratrol
SIRT1: sirtuin-1
Ten6: Tenovin-6
Z-FL: Z-FL-COCHO
