# Supplementary material for: Cysteine cathepsins control hepatic NF-κB-dependent inflammation via sirtuin-1 regulation
Source: Cell Death Dis. 2016 Nov 10;7(11):e2464–. doi: 10.1038/cddis.2016.368 (PMC5260902; doi:10.1038/cddis.2016.368)
Supplement: Supplementary Information [file cddis2016368x1.docx]

**SUPPLEMENTARY FIGURES**

**A**

**B**

**Figure S1. A.** **Inflammatory gene induction (MCP1 and IL6 mRNAs) during acute inflammation by LPS injection in mice lacking macrophages.** Mice were treated with liposomes (PBS or clodronate, 0,25mL/mouse) two days before LPS (1.0mg/kg) i.p. injection. Results are given as a mean ± S.D. of three independent experiments; * p < 0.001 vs Control, and ¶ p< 0.001 vs LPS. **B. Evaluation of KCs presence in liver tissue after liposomal clodronate injection**. Mice treated with CD-liposomes were compared with PBS-liposomes mice. F4/80 (brown) is present in PBS-mice, while CD-mice show a total decrease of this macrophage marker.

**Figure S2. Gene array displaying the effect of CTSB inhibition on inflammation and fibrosis markers in liver samples of mice with NASH.**

NASH was induced by feeding mice a HFCD diet for 8 weeks; CA-074-treated mice received a daily dose (10mg/kg, i.p.) for the last week. The array was performed with 6 mice per group. Results are given as a mean ± S.D., n=6, *p<0.1 vs Control, #p<0.1 vs. HFCD-diet fed mice.
